# LXR-dependent enhancer activation regulates the temporal organization of the liver’s response to refeeding leading to lipogenic gene overshoot

**DOI:** 10.1371/journal.pbio.3002735

**Published:** 2024-09-12

**Authors:** Noga Korenfeld, Tali Gorbonos, Maria C. Romero Florian, Dan Rotaro, Dana Goldberg, Talia Radushkevitz-Frishman, Meital Charni-Natan, Meirav Bar-Shimon, Carolyn L. Cummins, Ido Goldstein

**Affiliations:** 1 Institute of Biochemistry, Food Science and Nutrition, The Robert H. Smith Faculty of Agriculture, Food and Environment, The Hebrew University of Jerusalem, Rehovot, Israel; 2 Department of Pharmaceutical Sciences, Leslie Dan Faculty of Pharmacy, University of Toronto, Toronto, ON, Canada; Columbia University, UNITED STATES OF AMERICA

## Abstract

Transitions between the fed and fasted state are common in mammals. The liver orchestrates adaptive responses to feeding/fasting by transcriptionally regulating metabolic pathways of energy usage and storage. Transcriptional and enhancer dynamics following cessation of fasting (refeeding) have not been explored. We examined the transcriptional and chromatin events occurring upon refeeding in mice, including kinetic behavior and molecular drivers. We found that the refeeding response is temporally organized with the early response focused on ramping up protein translation while the later stages of refeeding drive a bifurcated lipid synthesis program. While both the cholesterol biosynthesis and lipogenesis pathways were inhibited during fasting, most cholesterol biosynthesis genes returned to their basal levels upon refeeding while most lipogenesis genes markedly overshoot above pre-fasting levels. Gene knockout, enhancer dynamics, and ChIP-seq analyses revealed that lipogenic gene overshoot is dictated by LXRα. These findings from unbiased analyses unravel the mechanism behind the long-known phenomenon of refeeding fat overshoot.

## Abstract 2

Refeeding after a period of fasting induces a metabolic switch in the liver and can lead to a ’fat overshoot’, where fat mass surpasses pre-fasting levels. The study elucidates the kinetics of transcriptional and chromatin changes induced by refeeding, revealing a lipogenic overshoot program that is regulated by the transcription factor LXRα.

## Introduction

When food is readily accessible and its consumption is possible at will (ad libitum), most mammals will eat several meals during their wake time and fast for a few hours during their inactive phase. However, mammals are often faced with longer periods of fasting for reasons such as inaccessibility of food, illness, or voluntary fasting [1]. In both the fed and fasted states, bodily homeostasis is maintained due to metabolic adjustments aimed at preserving energy supply to cells and storage of excess energy. When food is consumed again after a period of fasting (i.e., refeeding), a metabolic switch occurs and tissues transition from frugal energy usage and the internal production of fuel to using energy available from food constituents and storage of excess energy in specialized molecules.

The liver plays a central role in maintaining homeostatic metabolic pathways important in both the fed and fasted states. During fasting, glycogen is broken down to supply glucose, and gluconeogenesis is enhanced to produce glucose from non-carbohydrate precursors. Additionally, fatty acid oxidation is augmented to produce ATP and supply precursors for the production of ketone bodies which are then used as an energy source for the brain and other tissues [2]. In the fed state, fatty acid oxidation is dampened and instead fatty acid synthesis is active. Synthesized fatty acids (together with glycerol) are used to produce triglycerides, the principal energy storage molecule in mammals. The pathways of fatty acid and triglyceride synthesis in the liver are termed “de novo lipogenesis” or simply “lipogenesis” [3,4]. Another major biosynthetic pathway active during the fed state is cholesterol biosynthesis [5]. Cholesterol serves as a constituent of membranes, lipoproteins and is the initial substrate for the synthesis of bile acids, steroids, and certain vitamins. Both lipogenesis and cholesterol biosynthesis require acetyl CoA as a precursor. Several metabolic pathways converge to acetyl CoA and two were shown to be important in supporting lipogenesis and cholesterol biosynthesis: the glycolytic production of pyruvate (which is eventually converted to citrate and then to acetyl CoA) and the production of acetyl CoA from acetate (acetyl CoA production from acetate is considered to be minor in physiological conditions) [6,7]. In addition to precursors, lipogenesis and cholesterol biosynthesis require NADPH which is supplied by malic enzyme activity as well as by enzymes in the pentose phosphate pathway [4,8].

Both hepatic lipogenesis and cholesterol synthesis are heavily regulated transcriptionally with dedicated transcriptional programs activating them in the fed state [5,9–12]. These programs include induction of genes encoding enzymes, transporters and carriers participating in these 2 anabolic pathways (for brevity, we term these genes and their encoded products “lipogenic genes” or “cholesterol biosynthesis genes”). Several transcription factors (TFs) were reported to govern hepatic induction of these genes under fed conditions. Two central TFs regulating lipid synthesis are members of the sterol regulatory element-binding protein (SREBP) family: SREBP1c (encoded from *Srebf1*) and SREBP2 (encoded from *Srebf2*). The activity of these TFs is regulated by proteolytic cleavage occurring in the Golgi. Following cleavage, SREBPs enter the nucleus, bind their DNA recognition motif, and induce gene transcription. The activation of SREBPs is controlled by cholesterol whereby cholesterol inhibits SREBP2 activation. SREBP1c is also inhibited by cholesterol but it is commonly accepted that the major regulation on SREBP1c activity is feeding-dependent whereby insulin levels rising in the fed state robustly activate SREBP1c [13]. Several studies using gene knockout techniques showed that SREBP1c mostly induces lipogenesis genes while SREBP2 induces cholesterol biosynthesis genes [11,12].

Another critical TF family regulating lipogenesis is the liver X receptor (LXR) family, composed of 2 members: LXRα (encoded from *Nr1h3*) and LXRβ (encoded from *Nr1h2*). LXRα is considered the principal LXR in hepatocytes and the major member responsible for lipogenesis (although in the absence of LXRα, LXRβ partially compensates for it [14]). LXRs induce many lipogenic genes and their deletion severely impairs lipid homeostasis [15–18]. Part of the positive effect of LXRs on lipogenesis is indirect, through induction of *Srebf1* and the resulting increase in SREBP1c levels [19–22].

The TF termed carbohydrate response element-binding protein (ChREBP) also supports lipogenesis, partly due to activation of glycolysis which supplies lipogenic precursors [23]. Similar to SREBP1c, ChREBP mRNA levels are also induced by LXR [24,25]. In addition to those mentioned above, other TFs were reported to regulate certain aspects of lipogenesis and cholesterol biosynthesis: thyroid hormone receptor (ThR) [26], upstream stimulatory factor 1 (USF-1) [27,28], X-box binding protein 1 (XBP-1) [29], and liver receptor homolog 1 (LRH-1) [30]. In addition to TFs activating lipogenesis, the BCL6 TF was shown to repress lipid catabolism in the fed state [31].

TFs regulate gene expression by binding to DNA regulatory elements (enhancers and promoter-proximal regions) and initiate a series of events resulting in enhancer activation followed by increased rate of gene transcription by RNA polymerase II (i.e., gene induction). These events include TF-dependent recruitment of co-activators, histone modifying enzymes, and chromatin remodelers [32]. It has become clear from many studies that enhancer activity in fully differentiated cells is altered by various hormonal and metabolic cues. This is beautifully exemplified in hepatocytes whose enhancers are dynamically activated by a myriad of cues in a TF-dependent manner [17,33–37].

The transcriptional regulation and underlying enhancer dynamics of the fasted state has previously been studied, leading to meaningful insights into liver biology and regulation of metabolic pathways [38]. In stark contrast, the response to refeeding has received considerably less attention and the refeeding enhancer landscape, its dynamics and kinetics after cessation of fasting have not been explored. Moreover, the ad libitum fed state and refed state are considered synonymous and most studies (apart from a few exceptions [39,40]) use either ad libitum fed or refed states to represent a “fed” state. Herein, we aimed to inspect the transcriptional and chromatin events occurring upon refeeding, their kinetic behavior and their molecular drivers. We found distinct, temporally organized transcriptional programs occurring upon refeeding with an early wave of transcription followed by a later wave. These programs were driven by enhancer activation that also showed kinetic behavior. Using unbiased genome-wide approaches and gene knockout models, we show that a lipogenic gene program is part of the second wave of transcription and is directly regulated by LXRα.

## Results

### Refeeding gene regulation distinctly diverges from the basal ad libitum state

**Fig 1:**
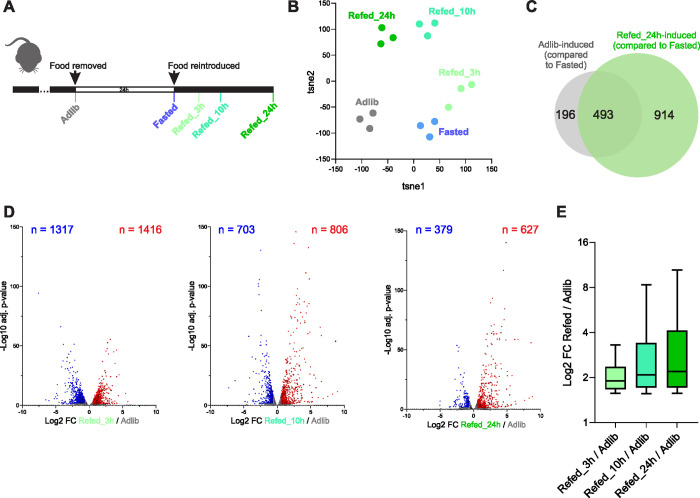
Gene expression upon refeeding distinctly diverges from the basal ad libitum state. (**A**) Experimental design—the different groups were collected at different food availability states; animals from the Adlib group did not experience fasting prior to collection. The Fasted group was collected at the end of a 24 h fasting bout. The Refed groups were collected 3, 10, and 24 h following reintroduction of food. (**B**) The global transcriptomic similarity between replicates and experimental groups was measured by a t-SNE analysis. Biological replicates cluster closely to each other, showing high transcriptomic similarity and attesting to the technical quality of the experiment. The Refed groups notably diverge from the Adlib group as well as from each other, pointing to marked differences in gene expression in the Refed groups compared to Adlib and to kinetic progression of the transcriptomic response to refeeding. (**C**) Evaluation of Refed-induced genes vs. Adlib-induced genes (both compared to the Fasted group) shows a distinct and nonoverlapping set of induced genes in the 2 groups. A gene was considered differentially regulated compared to Fasted if it passed 2 cutoffs determined by DESeq2: FC ≥1.5 and adj. *p*-value ≤0.05. These cutoffs are consistent throughout the study. (**D**) A direct pairwise comparison between the Adlib and each Refed time point uncovers thousands of genes differentially regulated upon refeeding, showing that the transcriptomes of the Refed and Adlib conditions are far from identical. (**E**) The FC values of refeeding-induced genes in each Refed time point were plotted (compared to Adlib; i.e., the genes marked in red in panel D). The highest fold induction is observed in later Refed time points (10 and 24 h). FC, fold change; t-SNE, t-distributed stochastic neighbor embedding.

We aimed to gain a better understanding of the hepatic transcriptional response to refeeding and its kinetic progression from early to later time points. Thus, we designed an experiment in which control mice had ad libitum access to food while all other groups were fasted for a period of 24 h. We chose to fast mice for 24 h because this length of fasting was repeatedly shown to lead to maximal fasting-dependent gene regulation and to optimally manifest the metabolic attributes of fasting (gluconeogenesis, glycogen breakdown, fatty acid oxidation, ketogenesis, etc.) [33,41,42]. One group of mice was euthanized at the end of the fasting period while the other groups had food reintroduced into the cage. Then, mice were euthanized at 3 different time points (3 h, 10 h, and 24 h) following refeeding. The groups were termed Adlib, Fasted, Refed_3h, Refed_10h, and Refed_24h, respectively (Fig 1A). The hepatic transcriptome of all groups was profiled via RNA-seq. To broadly assess the difference between the transcriptomes of different groups, we performed a t-distributed stochastic neighbor embedding (tSNE) analysis. This showed a gradual departure from the basal gene expression pattern (Adlib) first to the Fasted state and then to the different Refed stages. Surprisingly, the Refed states were noticeably distinct from the basal Adlib state and the liver gene expression pattern did not return to the basal state even in animals who had unlimited access to food for 24 h following fasting (Fig 1B). To further show the difference between the Adlib and Refed states, we performed pairwise differential gene expression analysis and defined genes whose expression is higher in the Adlib condition compared to the Fasted condition. We then compared them to the genes whose expression is higher in the Refed_24h condition compared to the Fasted condition. Adlib and Refed states are considered similar conditions because they are both “fed” states in which energy from food is readily available. If indeed the Adlib and Refed_24h states are very similar to each other, we would expect the transcriptional program of these 2 conditions in comparison to Fasted to largely overlap. We also note that this is the only refeeding time point that matches in circadian time that of the fasted samples. However, we found only partial overlap between the 2 gene groups where only 35% of genes induced in the Refed_24h/Fasted comparison were also induced in the Adlib/Fasted comparison (Fig 1C and S1 Table; in all analyses throughout the study, a gene was considered differentially regulated if it passed 2 cutoffs: fold change (FC) ≥1.5 and adj. *p*-value ≤0.05). We then sought to directly measure differential gene expression between the Adlib and Refed conditions; 2 biological conditions commonly perceived as interchangeable. We found that 2,209 genes were induced in at least 1 refeeding time point as compared to the Adlib state (S1 Table). While the early refed time point led to a higher number of induced genes (Fig 1D), the potency of gene induction, as measured by FC, was higher in later refeeding time points (Fig 1E). Collectively, these data show that refed mice have a fundamentally different milieu of expressed genes at all measured refed time points compared to ad libitum fed mice.

### Refeeding gene regulation is temporally organized

While pairwise comparisons are useful in obtaining discrete gene lists with distinct FC values, they fail to encompass dataset-wide gene expression patterns and trends. In order to expose these complex patterns, we created 2 gene lists for downstream analyses which represent 2 opposite biological responses: fasting-induced genes and fasting-repressed genes. A list of fasting-induced genes was created by extracting all genes showing lower expression in either of the fed states (Adlib, Refed_3h, Refed_10h, or Refed_24h) as compared to the Fasted state (*n* = 2,312; S2 Table). To generate the reciprocal type of regulation—fasting-repressed genes, we collected all genes showing higher expression in either of the fed states as compared to the Fasted state (*n* = 2,825; S2 Table). Then, each gene list was put through a clustering analysis to reveal major gene expression patterns and trends.

**Fig 2:**
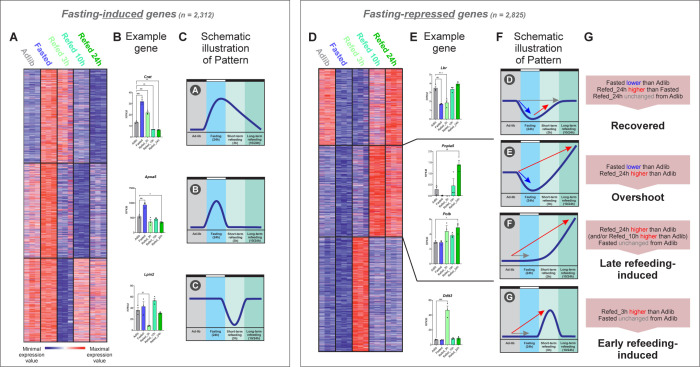
Induction of several distinct gene expression programs following refeeding. (**A**) k-means clustering of fasting-induced genes (*n* = 2,312; k = 3) shows 3 major gene expression patterns. Blue: minimum expression value of the gene. Red: maximum expression value of each gene (minimum and maximum values of each gene are set independently to other genes). (**B**) The normalized read values are shown for a representative gene from each cluster. Conditions in which gene expression was different from Adlib in a statistically significant manner are marked with asterisks. Numerical values for this panel are detailed in S1 Data. (**C**) Schematic illustration of patterns recovered in clustering analysis (Fig 2A): *Pattern A*: Fasting-induced genes whose expression wanes slowly upon refeeding. *Pattern B*: Fasting-induced genes going back to their basal levels quickly upon refeeding. *Pattern C*: Genes with similar expression between Adlib and Fasted but quickly repressed upon refeeding and then go back to basal levels at later refeeding time points. (**D**) k-means clustering of fasting-repressed genes (*n* = 2,825; k = 3). Blue: minimum expression value of the gene. Red: maximum expression value of each gene (minimum and maximum values of each gene are set independently to other genes). (**E**) Careful inspection of the second cluster revealed it represents 2 gene expression patterns. The normalized read values are shown for a representative gene from each pattern. Conditions in which gene expression was different from Adlib in a statistically significant manner are marked with asterisks. Numerical values for this panel are detailed in S1 Data. (**F**) Schematic illustration of patterns recovered in clustering analysis (Fig 2D): *Pattern D*: Fasting-repressed genes recovering to their Adlib basal levels upon refeeding. *Pattern E*: Genes repressed by fasting and upon refeeding overshoot above their basal levels. *Pattern F*: Genes with similar expression between Adlib and Fasted but potently induced in later refeeding time points. *Pattern G*: Genes quickly and transiently induced in Refed_3h. (**G**) Definitions of the genes belonging to each pattern based on the detailed cutoffs. These cutoffs are schematically represented by arrows in panel F: Gray arrow indicates no statistically significant change between 2 conditions, red arrow indicates gene induction and blue arrow indicates gene repression.—All individual data points are presented ± SD; *P ≤ 0.05, **P ≤ 0.01, ***P ≤ 0.001, ****P ≤ 0.0001 by ordinary one-way ANOVA followed by Dunnett’s post hoc analysis. RPKM, reads per kilobase per million reads.

Clustering of the fasting-induced genes revealed 3 clusters consisting of 3 gene expression patterns (Fig 2A). The first cluster showed fasting-induced genes whose levels remain high after 3 h of refeeding and only return to basal levels after 10 or 24 h of refeeding (Pattern A). Pathway enrichment analysis showed many genes from this cluster participate in lipid catabolism, fatty acid oxidation, and ketogenesis. This aligns with the well-known induction of these pathways during fasting [43]. In the second cluster, fasting-induced genes quickly reversed to their Adlib levels with repression evident as early as 3 h following refeeding (Pattern B). Enriched pathways in this cluster were related to signal transduction and phosphorylation. Interestingly, in the third cluster we found a group of genes whose levels do not overtly change between the Adlib and Fasted states but is markedly reduced following 3 h of refeeding and going back to basal levels at later refeeding time points (Pattern C). Genes from this cluster belonged to various signaling pathways. A representative gene from each cluster is depicted (Fig 2B and S1 Data) and a schematic illustration of each pattern is shown in Fig 2C. The full gene lists and enriched pathways of each pattern are detailed in S2 Table.

Clustering of fasting-repressed genes revealed 3 major clusters (Fig 2D). Upon careful inspection, the 3 clusters represented 4 gene expression patterns (see gene examples and schematic illustrations in Fig 2E and 2F and S1 Data). The first cluster showed gene repression upon fasting which did not resolve 3 h after refeeding. Only 24 h after refeeding did gene expression recover and return to its basal Adlib levels (Pattern D). This intuitive pattern was expected and is the one most chiefly considered in the literature [44]. The second cluster revealed 2 intriguing patterns where following fasting-mediated gene repression, genes were strongly induced in later refeeding time points to a level higher than the basal state (Pattern E). Within the same cluster, it appears that the expression of some genes does not differ between the Adlib and Fasted conditions, but does show overt induction upon refeeding (Pattern F). The third cluster was also unexpected with a clear pattern of immediate gene induction following 3 h of refeeding which wanes almost completely after 10 h of refeeding (Pattern G). Taken together, these findings reveal a dynamic transcriptional response to refeeding with clear kinetics whereby gene induction following refeeding is partitioned to an early response and a late response. Also, refeeding often leads to strong gene induction higher than the basal ad libitum state.

Because the Refed_3h and Refed_10h conditions were collected at different time points during the day, it is conceivable that some genes induced in these conditions are induced not due to refeeding but rather due the effect of the circadian clock which significantly alters liver gene expression throughout the day [45]. The Adlib, Fasted, and Refed_24h groups were collected at zeitgeber time 1 (ZT1) while the Refed_3h and Refed_10h groups were collected at ZT4 and ZT11, respectively. To test the contribution of rhythmic gene expression on these groups, we compared the genes induced in the Refed_3h or the Refed_10h condition (as compared to Adlib) to genes induced in ad libitum-fed mice whose transcriptome was profiled in ZT points similar to Refed_3h and Refed_10h [46]. There was very little overlap between rhythmic clock-controlled genes in the relevant time points and genes induced by refeeding in Refed_3h and Refed_10h (S1 Fig), suggesting that most of the genes revealed in our clustering analysis are genes responding to refeeding per se and not to the circadian clock.

To further explore the transcriptional events occurring upon refeeding, we considered the 4 refeeding patterns from Fig 2F (Patterns D, E, F, and G) and defined distinct inclusion criteria for each pattern. The criteria are based on fold change and statistical significance cutoffs designed for each pattern (Fig 2G). In Patterns F and G, the prominent change in gene expression is observed when comparing the Refed states to Adlib while the difference between the Adlib and Fasted states is mild or even nonexistent. Pattern G, termed “early refeeding induction,” was defined by cutoffs to represent significant induction in the Refed_3h as compared to Adlib but no induction compared to the other conditions. This resulted in 1,047 early-refed-induced genes and pathway enrichment analysis revealed that many of these genes participate in protein synthesis, ribosomal biogenesis, rRNA processing, etc. (S2 Table). This induction of genes related to protein synthesis presumably serves to support the previously reported increase in liver cell mass and hepatocyte proliferation following refeeding [47–50]. For Pattern F (late refeeding-induced genes), we used a similar approach in which genes were included if they were induced in Refed_10h or Refed_24h as compared to Adlib but were not induced in Fasted or Refed_3h compared to Adlib. This resulted in 747 refeeding-induced genes enriched with similar pathways as early refeeding-induced genes (S2 Table).

The 2 other gene expression patterns (Patterns D and E) were characterized by repression during fasting, compared to the Adlib state. In Pattern D, fasting-repressed genes recover and go back to their basal, pre-fasting expression levels. In contrast, genes in Pattern E showed an overshoot pattern where expression is reduced during fasting and upon refeeding, it markedly exceeds basal levels. Again, to strictly differentiate between “recovered” genes and “overshoot” genes, we used distinct cutoffs. Because the Refed_10h and the Refed_24h gene expression trends were similar, we focused only on the Refed_24h time point. Genes repressed by fasting compared to both Adlib and Refed_24h that also show no higher expression in Refed_24h compared to Adlib were defined as recovered (*n* = 419; S2 Table). Genes both repressed by fasting compared to Adlib and induced by refeeding (again, compared to Adlib) were determined as overshoot genes (*n* = 74; S2 Table).

### Lipogenic genes and cholesterol biosynthesis genes are differentially regulated during refeeding

Given their different expression patterns, we hypothesized the 2 gene groups—“recovered” and “overshoot”—may have different functions and therefore we performed pathway enrichment analysis for each group. A prominently enriched pathway in the recovered group was cholesterol biosynthesis, in line with the known repression of cholesterol biosynthesis genes during fasting [5]. In the overshoot group, cholesterol biosynthesis was also enriched but in addition, the pathways for lipogenesis, fatty acid synthesis, triglyceride synthesis, glycolysis, and the pentose phosphate pathway were enriched as well (S2 Table).

**Fig 3:**
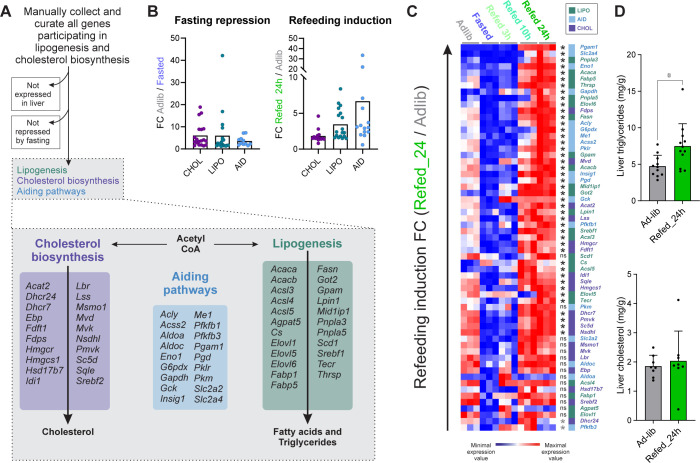
Upon refeeding most cholesterol biosynthesis genes recover to pre-fasting levels while lipogenesis genes overshoot. (**A**) To faithfully define genes whose encoded proteins participate in lipid synthesis or aiding pathways, we first collected all relevant genes from the literature. We then applied 2 filtering steps in which all genes not expressed in liver (RPKM below 1) and were not repressed by fasting were excluded. The 3 groups were abbreviated as follows: LIPO–lipogenesis genes; CHOL–cholesterol biosynthesis genes; AID–genes from pathways needed to aid both lipogenesis and cholesterol biosynthesis (e.g., to produce acetyl-CoA or replenish NADPH levels). For further details and full gene lists, see S3 Table. (**B**) The extent to which LIPO, CHOL, and AID genes are repressed by fasting (Adlib/Fasted) or induced by refeeding (Refed_24h/Adlib) was measured. While average fasting repression FC was similar between LIPO and CHOL genes, refeeding induction FC was higher in LIPO genes. Each point represents the FC of a single gene. Numerical values for this panel are detailed in S1 Data. (**C**) The expression level of all CHOL, LIPO, and AID genes is presented, showing robust overshoot induction of many LIPO and AID genes following refeeding with most CHOL genes showing a recovered pattern. All genes from panel A are shown and were sorted based on refeeding-induction FC. Genes induced in Refed_24h compared to Adlib in a statistically significant manner are marked with a black asterisk while those that did not pass the adj. *p*-value cutoff are marked by “ns.” Genes significantly repressed in Refed_24h compared to Adlib are marked with a gray asterisk (adj. *p*-values were determined by DESeq2). (**D**) Liver triglycerides and total cholesterol were quantified, showing increased liver triglycerides following refeeding. All individual data points are presented ± SD; *P ≤ 0.05, **P ≤ 0.01, ***P ≤ 0.001, ****P ≤ 0.0001 by two-tailed unpaired *t* test. One significant outlier was removed based on the ROUT method, Q = 1%. Numerical values for this panel are detailed in S1 Data. FC, fold change; RPKM, reads per kilobase per million reads.

These results suggest that while most cholesterol synthesis genes only go back to their basal level after refeeding, genes related to other lipid metabolic pathways overshoot following refeeding. To examine this possibility, we manually collected and curated from the literature all genes related to cholesterol biosynthesis as well as lipogenesis and divided them into groups: The CHOL group consists of all genes previously shown to participate in the cholesterol biosynthesis pathway. The LIPO group consists of all genes shown to participate in lipogenesis (fatty acid synthesis, fatty acid elongation, and triglyceride synthesis). Many genes are intimately related to both cholesterol biosynthesis and lipogenesis because they aid and support both pathways in various ways. For example, genes contributing to the formation of acetyl-CoA (the precursor for both biosynthetic pathways) and genes replenishing NADPH, a cofactor needed for both cholesterol biosynthesis and lipogenesis pathways. We collected all these genes in a group termed AID. After obtaining the 3 lists, we excluded all genes not expressed in liver as well as genes not repressed by fasting as compared to any of the fed conditions (S2 Table). After these stringent filtering steps, we were left with 61 genes belonging to either the LIPO, CHOL, or AID groups (Fig 3A and S3 Table).

To test for different regulatory modes, we first assessed whether CHOL and LIPO genes are repressed to different strengths during fasting. Comparison of FC values (Fasted compared to Adlib) showed no difference in repression potency between LIPO and CHOL genes. Then, we aimed to assess if refeeding induction values differ between groups. Here, and throughout the rest of the text, the term “refeeding induction” refers to an increase in the Refed_24h condition as compared to Adlib. We compared the FC values of Refed_24h to Adlib and found a significant difference between groups: most CHOL genes showed little-to-no refeeding induction while many LIPO and AID genes were robustly induced by refeeding as compared to the basal, Adlib condition (Fig 3B and S1 Data). To represent this visually, we plotted the expression values of all 61 genes across all conditions. This shows that most (although not all) CHOL genes show a recovered pattern while LIPO genes mostly overshoot (Fig 3C). AID genes largely followed the LIPO pattern of expression with high refeeding induction above Adlib levels (Fig 3C). The common transcriptional pattern between LIPO and AID genes suggests a mutual transcriptional regulator and implies that the induction of AID genes serves to support lipogenesis. Indeed, many AID genes are commonly considered to facilitate lipogenesis much more prominently than cholesterol biosynthesis (e.g., *Acly*, *Me1*).

To test whether these gene expression changes alter hepatic lipid levels, we quantified liver triglyceride and cholesterol levels. In accordance with LIPO gene overshoot, triglyceride levels were increased following refeeding as compared to Adlib. In contrast, there was no change in liver cholesterol levels, aligning with the lack of cholesterol gene overshoot (Fig 3D and S1 Data). Plasma cholesterol and triglyceride levels were unchanged between Adlib and Refed_24h (S2A Fig and S1 Data). Collectively, these findings show that while both cholesterol biosynthesis and lipogenesis pathways are similarly repressed by fasting, they are regulated in a starkly different manner following refeeding with many LIPO and AID genes overshooting above pre-fasting levels. Similarly to LIPO and AID gene expression, liver triglycerides levels also overshoot above basal levels following refeeding.

The overshoot phenomenon is evident 24 h following refeeding. To test if it persists even longer, we modified the fasting-refeeding experiment and examined longer refeeding periods—in addition to the Refed_24h time point, we collected livers 72 h and 1 week after the reintroduction of food. LIPO and AID genes showed overshoot expression upon 24 h of refeeding, as expected. Interestingly, for some genes the overshoot phenomenon lingered also in the Refed_72h group where gene expression remained higher than Adlib. By 1 week after refeeding, all genes returned to their basal expression (S2B Fig and S1 Data). Accordingly, hepatic triglyceride content tended to be higher 24 and 72 h after refeeding (although it did not reach statistical significance; S2C Fig and S1 Data). Therefore, we found that gene and fat overshoot upon refeeding lasts for 3 days after reintroduction of food.

Our experiments included 24 h of fasting prior to refeeding. This period of fasting extends beyond the mice’s inactive phase through which mice fast voluntarily for several hours. Shorter periods of fasting of around 8 h during the inactive phase are considered mild and do not lead to maximal glycogen depletion, ketonemia, weight loss, and other parameters of the fasting response [33,41]. We aimed to test if periods of fasting closer to voluntarily overnight fasting lead to overshoot upon refeeding. Therefore, we performed a fasting-refeeding experiment where mice fasted for only 8 h followed by 24 or 72 h of refeeding. Under these conditions, genes did not overshoot (S2D Fig and S1 Data). Thus, short-term fasting periods during the inactive phase are not followed by gene overshoot.

### Refeeding increases chromatin accessibility and leads to enhancer overshoot

**Fig 4:**
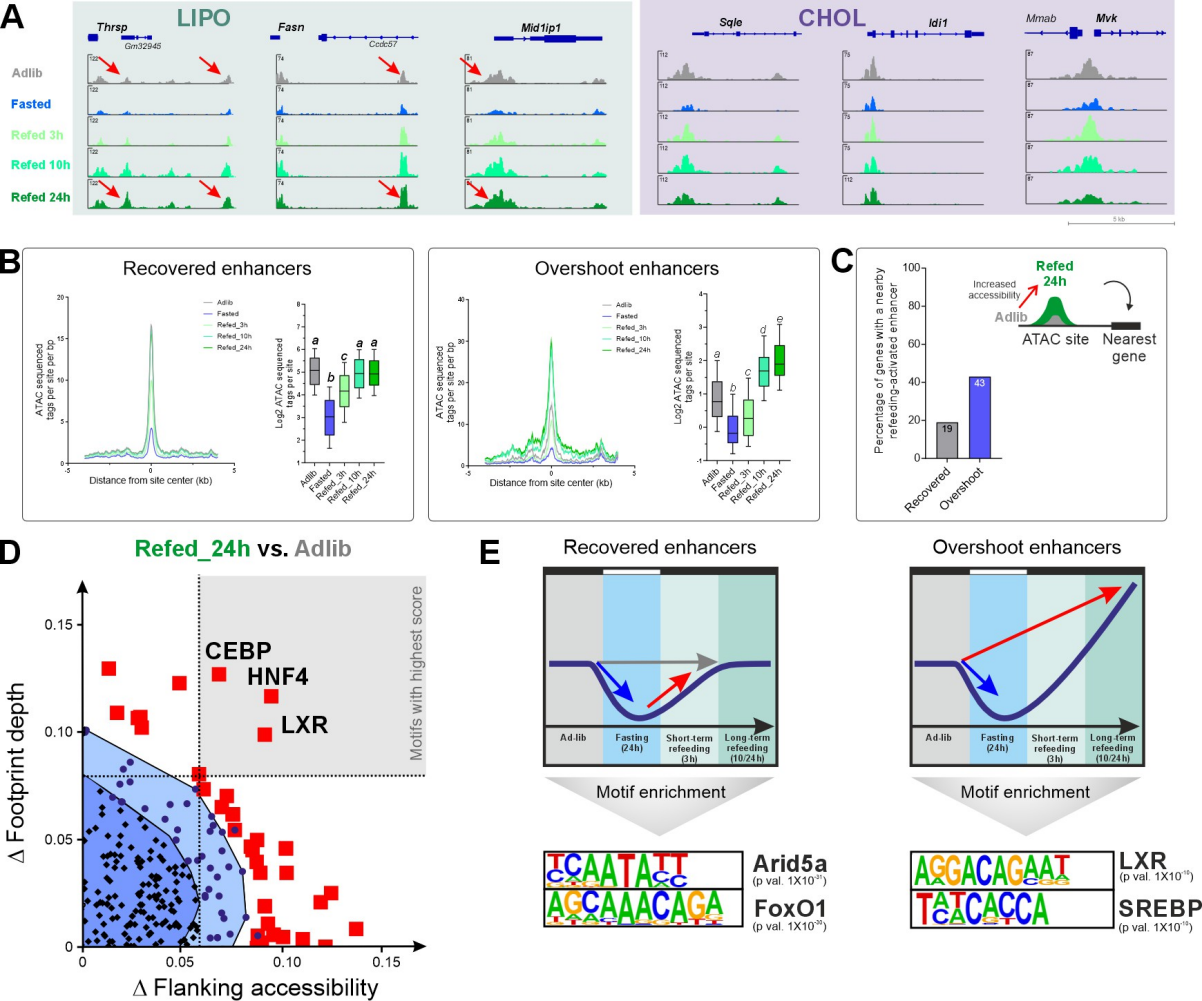
Refeeding leads to enhancer overshoot with increased LXR footprint and enrichment of the LXR motif. (**A**) The chromatin accessibility at loci of LIPO and CHOL genes are depicted. All loci show regions with fasting-dependent decrease in accessibility but only LIPO loci show an overshoot pattern in which the accessibility of Refed_10h/24h is prominently higher than Adlib. A selected replicate from each group is presented. (**B**) Quantification of chromatin accessibility at overshoot and recovered enhancers reveals that accessibility of overshoot enhancers markedly surpasses Adlib levels following 10 h and 24 h of refeeding. In contrast, recovered enhancers do not go over Adlib levels. Lower case letters above each box represent statistical significance: Boxes with different letters are statistically significantly different as measured by ordinary one-way ANOVA followed by Holm–Sidak post hoc analysis. Recovered and overshoot enhancers were defined based on the same FC and *p*-value cutoffs as were genes. (**C**) The presence of a refeeding-activated enhancer next to overshoot and recovered genes was determined, showing that overshoot genes are more likely to have an adjacent refeeding-activated enhancer. (**D**) Bivariate Genomic Footprinting (BaGFoot) analysis reveals TFs predicted to be activated in refeeding-activated enhancers based on increased FPD (y-axis) and FA (x-axis). LXR is among the top 3 TFs predicted to be highly active at refeeding-activated enhancers with prominent increases in both FA and FPD (top right region, gray-shaded). (**E**) The top motifs enriched in each enhancer group are shown (the full list is presented in S4 Table), with LXR absent from recovered enhancers but highly enriched in overshoot enhancers. FA, flanking accessibility; FC, fold change; FPD, footprint depth; LXR, liver X receptor; TF, transcription factor.

Next, we aimed to reveal the transcriptional regulatory module driving the overshoot phenomenon observed in LIPO and AID genes. Several TFs were shown to regulate hepatic lipogenesis with the most highly documented TFs being SREBP1c, LXR, ChREBP, and ThR. Because any of these TFs (and others) could potentially contribute to gene overshoot, we wanted to tackle this question in a TF-unbiased manner. Thus, we profiled enhancer activity with the aim of predicting the TF leading to gene overshoot from enhancer activity data. It was repeatedly shown by us and others that dynamic changes in chromatin accessibility mostly reflect changes in enhancer activity whereby increased chromatin accessibility implies enhancer activation [33,51–55]. Therefore, we profiled all accessible regions in liver via ATAC-seq in all feeding/fasting conditions (Fig 1A). Most sites of open chromatin were distal from gene promoters (S3A Fig). Moreover, motif enrichment analysis showed that accessible sites are highly enriched with liver lineage determining factors (S3B Fig) [56], suggesting many of these sites are distal DNA regulatory elements, i.e., enhancers. Examining the loci of LIPO and CHOL genes showed that chromatin accessibility dynamics largely follows that of gene expression (Fig 4A). Indeed, while enhancer accessibility around CHOL genes was repressed by fasting and went back to basal levels upon refeeding, LIPO enhancers showed an “enhancer overshoot” pattern following refeeding in which enhancer accessibility was increased above basal levels (Fig 4A). To clearly differentiate between the 2 enhancer populations, we defined overshoot and recovered enhancers in the same manner as we did for gene groups (S4 Table). Quantification of accessibility at these 2 enhancer populations across all conditions show that recovered enhancers have reduced accessibility in fasting which is gradually resolved, until reaching full recovery in Refed_24h. Overshoot enhancers also show fasting-dependent reduction but significantly surpass basal levels at later refeeding time points (Fig 4B). To link the increase in accessibility to gene expression patterns, we mapped the nearest gene to each refeeding-activated enhancer (i.e., an enhancer whose accessibility increases in the Refed_24h condition above Adlib levels) and cross-referenced them with the overshoot and recovered genes defined in Fig 2G. We found that 43% of overshoot genes have such an enhancer proximal to them compared to only 19% of recovered genes (Fig 4C). This shows that overshoot genes are more likely to have a proximal overshoot enhancer and thus links enhancer activation patterns with nearby gene induction patterns.

To predict the TFs involved in overshoot-related enhancer activation, we employed an enhancer-wide approach termed BaGFoot [57] that measures 2 tell-tale signs left by TFs on chromatin: TF “footprint” and “flanking accessibility.” Increased TF activity and/or increased TF dwell time on chromatin is often accompanied by local protection of the TF motif from transposase cleavage, resulting in a deeper TF “footprint” [52]. Likewise, increased TF activity leads to recruitment of co-activators, histone modifying enzymes, and chromatin remodelers. These lead to enhancer activation which can be measured by increased accessibility around the motif (“flanking accessibility”). BaGFoot measures both footprint depth (FPD) and flanking accessibility (FA) of all known motifs at every accessible site found in our data set across the genome. Then, BaGFoot calculates how different the 2 values are between 2 experimental conditions. To predict TFs that are differently activated in refeeding compared to ad libitum, we had BaGFoot compare the Refed_24h chromatin state to the Adlib state. The further the TF’s value is from the origin, the more it is predicted to be highly activated above Adlib levels. TFs strongly predicted to be activated in refeeding are presented in Fig 4D (for the complete list, see S4 Table). Interestingly, the LXR motif was among the top TFs predicted to be activated in refeeding. In addition to LXR, other lipogenesis-related TFs were predicted to be activated, albeit to a much weaker extent (S4 Table). While the BaGFoot results pointed to LXR, because BaGFoot is a genome-wide analysis, it is unable to directly link LXR to specific overshoot enhancers. Therefore, we performed de novo motif enrichment analysis in overshoot and recovered enhancers to find TF motifs specifically enriched in each enhancer group. Motif enrichment analysis of overshoot enhancers uncovered a motif similar to the LXR half-site; the LXR half-site is AGGTCA followed by a 4-nucleotide spacer and the enriched motif we found is AGGACA followed by a spacer. Of note, enrichment of a half-site slightly diverging from the consensus is not entirely surprising as LXR was shown to have a wide binding preference and bind sequences diverging from the consensus [58,59]. In perfect agreement with the BaGFoot results, the enriched LXR half-site was the top-enriched motif in overshoot enhancers while it was not enriched in recovered enhancers (Fig 4E and S4 Table), suggesting that LXR plays a regulatory role in gene overshoot.

### LXR dictates gene and enhancer overshoot controlling lipogenesis

**Fig 5:**
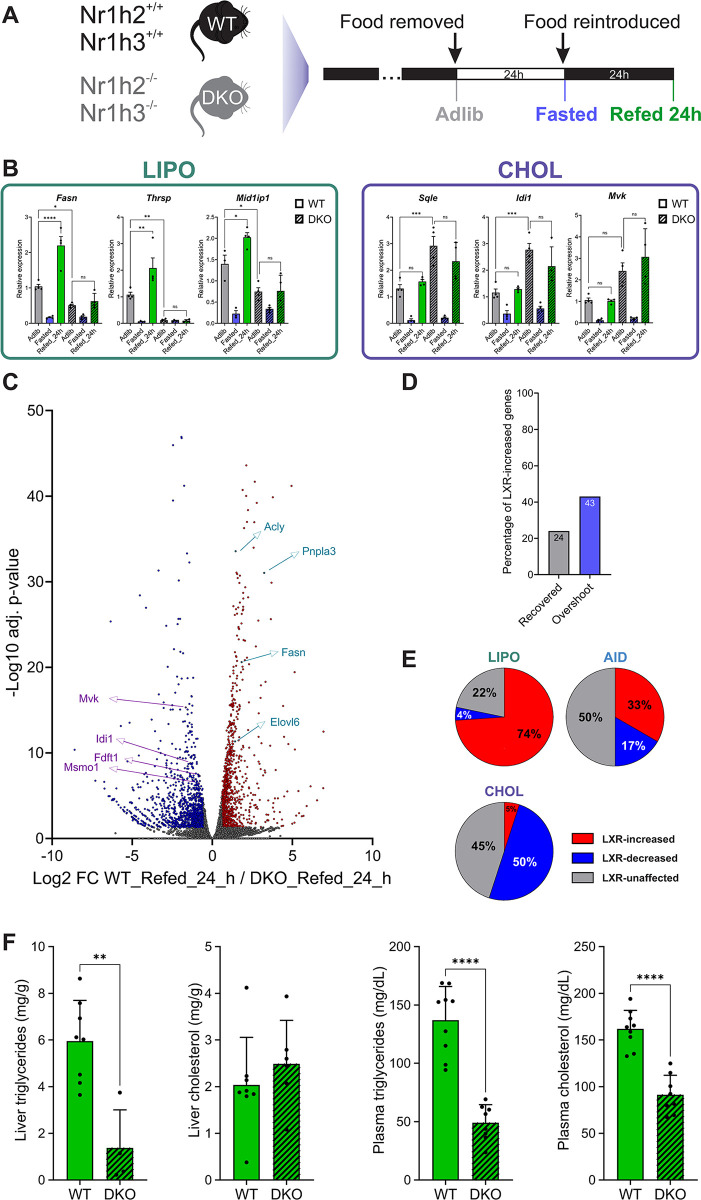
LXR governs bifurcation of lipid synthesis genes during refeeding and dictates gene overshoot. (**A**) Livers from WT and DKO mice for *Nr1h2* (LXRβ) and *Nr1h3* (LXRα) were collected at these conditions: ad libitum (Adlib), following 24 h of fasting (Fasted) or 24 h of fasting followed by 24 h of refeeding (Refed_24h). (**B**) The expression of representative LIPO, CHOL, and AID genes was measured via qPCR, revealing a divergent pattern of regulation by LXR which dictates overshoot of LIPO genes while repressing CHOL genes. Numerical values for this panel are detailed in S1 Data. (**C**) A direct pairwise comparison between WT and DKO mice shows 2,333 differentially regulated by LXR upon refeeding. The analysis shows a prominent role for LXR in increasing levels of LIPO genes while repressing CHOL genes during refeeding. (**D**) Overshoot and recovered genes whose expression is increased by LXR were determined, showing that overshoot genes are more likely to be increased by LXR as compared to recovered genes. (**E**) Examination of all genes in each group (LIPO, CHOL, and AID) shows that most LIPO genes are increased by LXR while half of CHOL genes are decreased by LXR. (**F**) Liver and plasma triglycerides and cholesterol were quantified in Refed_24h mice, showing decreased liver triglycerides, plasma triglycerides, and plasma cholesterol in the absence of LXR. Numerical values for this panel are detailed in S1 Data.—All individual data points are presented ± SD; *P ≤ 0.05, **P ≤ 0.01, ***P ≤ 0.001, ****P ≤ 0.0001 by ordinary one-way ANOVA followed by Holm–Sidak post hoc analysis (B) or by two-tailed unpaired *t* test (F). DKO, double knockout; LXR, liver X receptor; qPCR quantitative PCR; WT, wild type.

To test the possible role of LXR in gene overshoot, we repeated the Adlib-Fasted-Refed paradigm in mice where both LXRα and LXRβ were deleted (termed DKO, for double knockout) as well as in wild-type (WT) control mice (Figs 5A and S4A and S1 Data). We quantified gene expression and found that LIPO genes show the aforementioned fasting-dependent repression followed by a refeeding overshoot pattern in WT mice. In contrast, while fasting led to gene repression in DKO mice, refeeding only led to recovery from repression but there was no overshoot above Adlib levels. The fasting/refeeding-dependent regulation of CHOL genes remained intact in DKO mice. Notably, the expression of CHOL genes was basally higher in DKO mice compared to WT mice, regardless of fed/fasted status (Fig 5B and S1 Data). This shows that LXR drives refeeding gene overshoot of lipogenesis genes.

We examined the Refed_24h condition on a transcriptome-wide scale by performing RNA-seq on liver samples from WT and DKO mice refed for 24 h. We found 2,333 genes regulated by LXR with 1,140 genes increased in the presence of LXR and 1,193 decreased (Fig 5C and S5 Table). Focusing on the 2 gene groups defined before, we found that 43% of overshoot genes are also LXR-increased genes, compared to only 24% of recovered genes (Fig 5D). We then directly examined the effect of LXR specifically on lipid-related genes. Strikingly, 74% of LIPO genes were increased with LXR and only 1 gene decreased. In stark contrast, while only 1 CHOL gene was increased with LXR, 50% of CHOL genes were decreased. As with other comparisons, AID genes largely followed the pattern of LIPO genes (Fig 5E and S5 Table). To explore this from a different angle, we analyzed liver RNA-seq data from mice treated with GW3965, an LXR agonist [60]. Out of 63 GW3965-induced genes, 8 genes belonged to the LIPO group, 2 to the AID group, and none to the CHOL group (S5 Table). These results strongly portray a bifurcated role for LXR during refeeding—it enhances lipogenesis genes while repressing cholesterol synthesis genes. As suggested by gene expression data, liver triglycerides were markedly reduced in DKO_Refed_24h mice as compared to WT_Refed_24h mice. However, liver cholesterol levels remained unchanged in DKO mice on a chow diet (without cholesterol). Liver cholesterol levels are affected by several processes: hepatocyte cholesterol biosynthesis, bile acid synthesis, cholesterol excretion, and lipoprotein intake/excretion. Thus, total hepatocyte cholesterol levels represent the sum of all processes mentioned above and the cholesterol biosynthesis aspect exerted by LXR may be negligible. Indeed, a lack of effect of LXR on total liver cholesterol levels was previously reported [61]. Plasma triglyceride and cholesterol levels were both reduced in DKO_Refed_24h mice as previously reported [15,18], reflecting the known effects of LXR on lipoprotein metabolism [62] (Fig 5F and S1 Data). Taken together, these findings show that cholesterol biosynthesis genes tend to go back to their basal levels upon refeeding and be decreased by LXR. In contrast, lipogenesis and aiding genes tend to overshoot following refeeding in an LXR-dependent manner.

**Fig 6:**
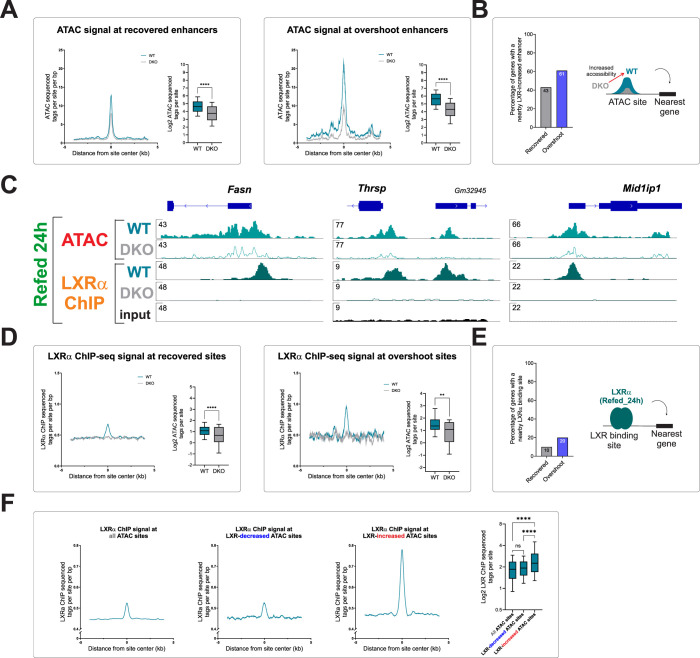
LXR directly binds and activates overshoot enhancers. (**A**) Quantification of chromatin accessibility at overshoot and recovered enhancers reveals that LXR increases accessibility of both enhancer types, with a stronger effect in overshoot enhancers. (**B**) The presence of an LXR-increased enhancer next to overshoot and recovered genes was determined, showing that overshoot genes are more likely to have an adjacent LXR-increased enhancer. (**C**) Loci of LIPO genes are depicted. All loci show an LXR-dependent increase in accessibility as well as prominent LXRα binding. The high signal-to-noise ratio in LXRα ChIP-seq signal between WT and DKO shows the high quality of the ChIP assay. (**D**) Quantification of LXRα binding at overshoot and recovered enhancers shows LXRα occupancy in both enhancer types, with stronger binding in overshoot enhancers. (**E**) The presence of an LXRα binding site next to overshoot and recovered genes was determined, showing that overshoot genes are more likely to have an adjacent LXRα binding site. (**F**) Quantification of LXRα binding at LXR-regulated enhancers shows that only LXR-increased enhancers are characterized with elevated LXRα occupancy. In contrast, LXR-decreased enhancers show LXRα occupancy which is comparable to LXRα occupancy across all hepatic accessible regions.—*P ≤ 0.05, **P ≤ 0.01, ***P ≤ 0.001, ****P ≤ 0.0001 by a two-tailed, unpaired *t* test. ChIP, chromatin immunoprecipitation; DKO, double knockout; LXR, liver X receptor; WT, wild type.

To explore the effect of LXR on enhancer activity, we performed ATAC-seq on livers from WT and DKO mice following refeeding. Differential accessibility analysis revealed 12,567 sites with LXR-dependent differential accessibility of which, 8,503 showed increased accessibility in the presence of LXR and 4,064 showed decreased accessibility (S6 Table). The LXR motif was a highly enriched motif among LXR-increased enhancers (S4B Fig). We then measured accessibility across all overshoot and recovered enhancers. Compared with DKO mice, the accessibility at overshoot enhancers more than doubled in WT mice. A similar, yet weaker trend was found in recovered enhancers where WT mice showed a 1.6-fold increase in accessibility compared to DKO mice (Fig 6A). This shows that the presence of LXR markedly increases chromatin accessibility at overshoot enhancers, with a more modest effect on recovered enhancers. To associate LXR-increased enhancers to the 2 gene groups, we mapped the genes nearest to LXR-increased enhancers. We found that 61% of overshoot genes were proximal to an LXR-increased enhancer, compared to 45% of recovered genes (Fig 6B). Examples for such genes and their surrounding enhancers are shown in Fig 6C.

The effect of LXR on these enhancers could be direct, i.e., mediated by binding of LXR at these enhancers and promoting enhancer activation. Alternatively, LXR could indirectly affect these enhancers via the gene induction of SREBP1c or ChREBP, followed by binding of these TFs to the enhancers, leading to their activation. The motif enrichment analysis showing high enrichment of the LXR motif suggests that LXR binds many LXR-activated enhancers. To test this directly, we performed chromatin immunoprecipitation sequencing (ChIP-seq) to LXRα (the major liver LXR isoform) in Refed_24h livers. We found 838 sites bound by LXRα (S6 Table) and as expected, the LXR motif was the most highly enriched motif within these sites (S4C Fig). A total of 144 of LXR-increased genes had a proximal LXRα binding site compared with only 26 LXR-decreased genes (S6 Table). This implies that LXRα binding is mostly associated with gene induction rather than repression. LXRα occupancy was detected near overshoot genes, many of which are LIPO genes (e.g., Fig 6C and S6 Table). Given this observation, we quantified LXRα binding at overshoot enhancers and found significant LXRα occupancy at overshoot enhancers (as well as in recovered enhancers, but to a much lesser extent, Fig 6D). Also, overshoot genes were more likely to reside next to an LXR-binding site compared to recovered genes (Fig 6E). To examine the possibility of increased LXRα occupancy following refeeding, we performed LXRα ChIP-PCR near several LIPO genes in the Adlib, Fasted, and Refed_24h conditions. LXRα occupancy was already detected prior to refeeding and did not significantly change between conditions (S4D Fig and S1 Data). Such constitutive LXRα occupancy is a previously reported characteristic of LXR [16,63] and aligns with known attributes of nuclear receptors that heterodimerize with RXR [64].

To examine the direct binding of LXRα in LXR-regulated enhancers genome wide, we quantified LXRα occupancy at all LXR-regulated enhancers. We found that LXRα occupancy at LXR-decreased enhancers was similar to the average LXRα occupancy levels across all enhancers. In stark contrast, LXRα occupancy was highly enriched at LXR-increased enhancers (Fig 6F). Therefore, LXRα binds near overshoot genes (as well as other LXR-increased genes), activates their enhancers, and promotes lipogenesis gene overshoot following refeeding.

Taken together, our findings in this study portray a temporally organized response to refeeding with distinct early and late responses. In the late response, LXRα binds and activates lipogenic enhancers, leading to gene overshoot in which lipogenic gene expression and liver triglyceride levels exceeds pre-fasting levels.

## Discussion

The reintroduction of food after prolonged fasting is associated with drastic metabolic adaptations. In the liver, increased glycolysis, cholesterol biosynthesis, and lipogenesis are observed. Dichotomic definitions of the “fasted” and “fed” states are often used to broadly describe 2 poles of metabolic states. As such, various states in which food is available and voluntarily consumed are all considered a “fed” state. With few exceptions [39,40], the ad libitum state and the refed state are widely considered interchangeable and only one of them is used in experimental setups to represent a fed state. Moreover, the kinetics of gene regulation upon refeeding and its underlying chromatin basis were not explored. Here, we found that refeeding following a fasting period is characterized by dramatic deviations from the basal ad libitum fed state in terms of enhancer and transcriptional programs. We found a bifurcation in the transcriptional programs dictating lipid synthesis with cholesterol biosynthesis genes showing similar gene expression levels in the ad libitum and refed states while lipogenic genes overshoot above basal levels upon refeeding.

The term “overshoot” was previously coined to describe a transient increase in fat mass following refeeding above pre-fasting levels in humans [65] and rodents [66]. The increased fat mass was accompanied by increased lipogenic enzymes in liver, which was postulated to be directed by thyroid hormone and glucocorticoids [66]. A transcriptional basis for the overshoot phenomenon was found when several lipogenic genes showed overshoot following 12 h of refeeding [67]. Later, a SREBP2-LXR-SREBP1c axis was suggested in which SREBP2 increases cholesterol levels thereby increasing the levels of LXR ligands which in turn induces SREBP1c levels and augments lipogenesis [68]. The latter finding joins the commonly accepted concept suggesting the role of LXR in lipogenic gene induction is solely achieved through its induction of *Srebf1* and the ensuing increase in SREBP1c activity. However, other evidence point to a broader role for LXR in lipogenesis which is partly independent of SREBP1c: agonist-mediated activation of LXR in *Srebf1*-deficient mice leads to partial induction of lipogenesis genes [69]. Also, while LXR DKO mice are resistant to diet-induced obesity and hepatic accumulation of triglycerides, *Srebf1* KO mice are not [15]. Our findings, summarized below, show that LXR plays a direct role in lipogenic gene overshoot, a role which extends beyond *Srebf1* induction: (**a**) The enhancers activated in the presence of LXR harbor the LXR binding motif, arguing against an indirect role in activating these enhancers. (**b**) LXRα abundantly binds at LXR-activated enhancers. (**c**) LXR binds in proximity to various LXR-increased genes, including lipogenic genes. While these findings do not diminish the role of SREBP1c in lipogenesis, they point to a direct role of LXR in binding and activating lipogenic enhancers. It is tempting to speculate that LXRα and SREBP1c cooperate on the chromatin template to synergistically induce lipogenic genes, a mechanism that was observed in fasting-related TFs [33,34].

In contrast to the role of LXR in promoting lipogenic gene induction, we found that LXR broadly represses cholesterol biosynthesis genes, fitting with earlier studies examining key cholesterol biosynthesis genes [18] and ATAC-seq experiments showing decreased accessibility near these genes [17].

We show that LXR activates thousands of enhancers throughout the genome. Together with a previous report [17], this places LXR as a central regulator of hepatic chromatin accessibility. LXR recruits co-activator proteins with capabilities to activate enhancers and increase chromatin accessibility [16]. Increased accessibility can facilitate the binding of additional TFs to the enhancers and the subsequent increase in gene transcription [56,58,70]. Therefore, the widespread role of LXR in hepatic enhancer accessibility which we show in this study suggests that LXR serves to assist the loading of other TFs to enhancers and together regulate gene expression.

We found that LXR robustly activates lipogenesis enhancers during refeeding. However, LXRα binding is also evident in the ad libitum and fasted states. Such constitutive LXRα occupancy is a previously reported characteristic of LXR [16,63]. There has been longstanding interest in the question of how LXR is activated in the fed state [71]. Tobin and colleagues have shown in rats that LXRα levels are increased with insulin [72]; Brown and Goldstein hypothesized that there may be an LXR ligand that is formed in the presence of insulin [19]; and there are numerous reports of LXRα activity changes based on posttranslational modifications that alter activity independent of ligand binding (phosphorylation [73], O-Glc-NAc-ylation [74]). In addition, posttranslational modifications can modulate coregulator recruitment [75]. Thus, it is likely that the mechanism by which LXRα is activated in refeeding is multifactorial.

In humans, fat overshoot was described after prolonged fasting periods [65]. Indeed, our results in mice suggest that only prolonged fasting leads to overshoot while shorter fasting periods during the inactive phase, during which mice are mostly sleeping, are not followed by overshoot upon refeeding.

In summary, our findings reveal a previously unknown temporal organization of the refeeding response with distinct kinetic patterns. We show that the early response to refeeding is mostly focused on a burst of transcriptional programs driving cell cycle progression, ribosomal biogenesis, and protein translation. This fits well with prior reports demonstrating increased cell proliferation and heightened ribosomal activity reported following refeeding [47–50]. The later stages of refeeding are dominated by a lipid-synthesis gene signature, although translation and ribosomal biogenesis programs are still at play. This lipid synthesis program is bifurcated with cholesterol biosynthesis genes and lipogenesis genes differentially regulated. The expression of cholesterol biosynthesis is constitutively inhibited by LXR and their levels mostly return to their pre-fasting levels. In contrast, LXRα potently induces lipogenic genes to an extent higher than pre-fasting levels by binding and activating their enhancers.

## Methods

### Animals

Male, 8 weeks old mice (C57BL/6J, Envigo) were acclimated for 1 week and randomly assigned to one of 5 groups (6 mice per group). The experiment started after 1 week of acclimation. The Adlib group had ad libitum access to food (regular chow diet, Teklad TD2018) and water throughout the experiment. For the rest of the groups, food was removed at the beginning of the inactive phase (shortly after lights on, ZT1). For the Refed groups, food was put back in the cage 24 h or 8 h later (as indicated in text) while the fasted group was anesthetized and euthanized. The Refed groups were euthanized 3 h, 10 h, 24 h, 72 h, and 1 week after food reintroduction. At each group endpoint, mice were anesthetized and euthanized (ketamine:xylazine 30:6 mg/ml) and the liver was excised.

For the studies using LXR DKO mice, 8- to 12-week-old male WT and LXRαβ-/- mice on a C57BL/6 background (a gift from Dr. David J Mangelsdorf, UT Southwestern, Dallas, Texas, United States of America) were bred in house. Mice were placed into one of 3 feeding groups: (a) ad libitum (regular chow diet, Teklad TD2016S); (b) fasted for 24 h; or (c) fasted for 24 h followed by a refeeding period of 24 h before humane euthanasia by decapitation at ZT1.

All animal procedures are compatible with the standards for the care and use of laboratory animals. The research has been approved by the Hebrew University of Jerusalem Institutional Animal Care and Use Committee; ethics approval numbers MD-22-17006 and MD-18-15596 (Jerusalem, Israel) or by the Faculty of Medicine and Pharmacy Advisory Committee on Animal Care at the University of Toronto; ethics approval number 20012519 (Toronto, ON, Canada).

### RNA preparation, reverse transcription, and quantitative PCR (qPCR)

Total RNA was isolated from liver pieces (30 mg) using a NucleoSpin kit (Macherey-Nagel cat# 740955.25) according to the manufacturer’s protocol. For qPCR, 1 μg of total RNA was reverse transcribed to generate cDNA (Quantabio cat# 76047–074). qPCR was performed using CFX96 or CFX Opus 384 thermal cycler instruments (Bio-Rad) using SYBR Green (Quantabio cat# 101414–276). Gene values were normalized to a housekeeping gene (*Rpl13*). The sequences of primers used in this study are:

*Rpl13*—Fwd: AGCCTACCAGAAAGTTTGCTTAC, Rev: GCTTCTTCTTCCGATAGTGCATC

*Fasn*—Fwd: GTGATAGCCGGTATGTCGGG, Rev: TAGAGCCCAGCCTTCCATCT

*Thrsp*—Fwd: CAGGAAATGACAGGGCAGGT, Rev: GATGCACTCAGAGGGAGACG

*Pnpla3*—Fwd: ACGGTGTCACCTTTCTACGG, Rev: CTCTCCCATCACCTTCACATCA

*Elovl6*—Fwd: GCAGAGAACACGTAGCGACT, Rev: CGCTTGTTCATCAGATGCCG

*Me1*—Fwd: GCCAAGGCAACAATTCCTACG, Rev: ACTGCAATTTTCAACGAAACGC

Acaca—Fwd: TCCACGAAAAGAGCTGACCT, Rev: ACTAAGGATGCTCCCCACCT

Acly—Fwd: TCGTCAACAAGATGAAGAAGGAGG, Rev: ATAAGATTTGGCTTCTTGGAGGTG

*Hmgcr*—Fwd: CGTCCAATTTGGCAGCTCAG, Rev: CCAGCGACTATGAGCGTGAA

*Sqle*—Fwd: TTGGTGGAGAGTGTGTGACC, Rev: TGGCGTAGATTGCAACGGAA

*Idi1*—Fwd: TTGAAGTACAGCTCTCCGCAC, Rev: CACATCTCCGCTAGAAGCTGAA

*Mvk*—Fwd: AACTTTCCTCCTGCTGCGAC, Rev: CTCTGTCACACGGGCAAACA

*Nr1h2*- Fwd: GTCCAGCTCTGCCTACATCG, Rev: TTGTAGTGGAAGCCCGAAGC

*Nr1h3*- Fwd: GATTAGGGTGGGGGTGACTG, Rev: CTGGAGCCCTGGACATTACC

### Triglyceride and cholesterol quantification

Plasma triglycerides (Wako) and cholesterol (Infinity, Thermo) were measured by colorimetric assays. Liver samples were cut (approximately 100 mg per piece), frozen in liquid nitrogen, and then stored at −80°C until extraction. Lipids were extracted from liver in chloroform/methanol (2:1, v/v) using the Folch separation technique [76]. Liver homogenates were then washed in 50 mM NaCl and centrifuged at 1500 × *g* for 30 min to separate the organic phase containing the lipids. Subsequent transfer and washing of organic phases in 0.36 M CaCl2/methanol was performed, followed by centrifugation at 1,500 × *g* for 10 min. Afterwards, the organic phases were brought to 5 ml with chloroform in a volumetric flask. Dried aliquots of standards and samples were redissolved in 10 μl of 1:1 chloroform/Triton X-100 overnight and assayed the following day for triglycerides (Infinity, Thermo Fisher Scientific or Sigma cat# MAK266) and cholesterol (Infinity, Thermo Fisher Scientific) using colorimetric reagents.

### Chromatin immunoprecipitation (ChIP)

ChIP was performed as previously described [31] with modifications: Liver pieces (150 mg) were cross-linked with phosphate-buffered saline (PBS) containing 2 mM disuccinimidyl glutarate (DSG, Santa Cruz Biotechnology, cat# sc-285455). Livers were homogenized with a Dounce homogenizer and rotated for 30 min at room temperature. Samples were centrifuged and the pellet was resuspended with PBS containing 1% formaldehyde (Electron Microscopy Sciences, cat# 15714) for further crosslinking. After 10 min, samples were quenched with 0.125 M glycine. Samples were then washed with PBS, resuspended in ChIP lysis buffer (0.5% SDS, 10 mM EDTA, 50 mM Tris-HCl (pH 8)) and sonicated (Bioruptor Plus, Diagenode) to release 100 to 1,000 bp fragments. Samples were diluted 1:5 with ChIP dilution buffer (170 mM NaCl, 17 mM Tris-HCl (pH 8), 1.2 mM EDTA, 1.1% Triton x-100, 0.01% SDS). Antibody against LXRα (R&D Systems cat# PP-PPZ0412-00, 4 μg per sample) was conjugated to magnetic beads (Sera-Mag, Merck, cat# GE17152104010150) for 2 h at 4°C. Chromatin was immunoprecipitated with antibody-bead conjugates for 16 h at 4°C. Immunocomplexes were washed sequentially with the following buffers: low salt buffer (0.01% SDS, 1% Triton x-100, 2 mM EDTA, 20 mM Tris-HCl (pH 8), 150 mM NaCl), high salt buffer (0.01% SDS, 1% Triton x-100, 2 mM EDTA, 20 mM Tris-HCl (pH 8), 500 mM NaCl), LiCl buffer (0.25M LiCl, 1% IGEPAL CA630, 1% deoxycholic acid, 1 mM EDTA, 10 mM Tris (pH 8.1)), and twice with TE buffer (10 mM Tris-HCl, 1 mM EDTA (pH 8)). Chromatin was eluted, de-crosslinked for 4 h at 65°C and deproteinized with proteinase K (Hy Labs, cat# EPR9016) for 1 h at 50°C. DNA was subsequently isolated using MinElute DNA purification kit (Qiagen cat# 20–28006). The sequences of primers used in ChIP-PCR are:

*Srebf1* –Fwd: CAGGCAACCATCCCCGAAA, Rev: ACAGAGCTTCCGGGATCAAAG

*Got2* –Fwd: ACCCCTTGATGTGGATTGGC, Rev: GTTACACAGGGCAGGTCAGT

*Fabp5* –Fwd: ACACTTGGAAACTCCTGACCC, Rev: CACCCCATACTGTGGGTAAACA

*Mid1ip*–Fwd: TATCAGGCGAGAGGCGGAG, Rev: GAGTAACACTCGCCCAACCC

### RNA-seq

Three replicates were randomly selected from each experimental group and processed for RNA-seq. For quality control of RNA yield and library synthesis products, the RNA ScreenTape and D1000 ScreenTape kits (both from Agilent Technologies), Qubit RNA HS Assay kit, and Qubit DNA HS Assay kit (both from Invitrogen) were used. mRNA libraries were prepared from 1 μg RNA using the KAPA Stranded mRNA-Seq Kit, with mRNA Capture Beads (KAPA Biosystems, cat# KK8421). The multiplex sample pool (1.6 pM including PhiX 1%) was loaded on NextSeq 500/550 High Output v2 kit (75 cycles) cartridge and loaded onto the NextSeq 500 System (Illumina), with 75 cycles and single-read sequencing conditions.

### ATAC-seq

ATAC-seq was performed as detailed in our freely accessible protocol [77] using 3 replicates for each group. Briefly, nuclei were isolated using a hypotonic buffer and Dounce homogenizer. Nuclei were tagmented using Tn5 transposase loaded with Illumina adapters. Tagmented DNA was PCR-amplified with sample-specific indices. The resulting library was size-selected to DNA fragments of 150 to 800 nt. The multiplex sample pool (1.6 pM including PhiX 1%) was loaded on NextSeq 500/550 High Output v2 kit (75 cycles) cartridge and loaded onto the NextSeq 500 System with 75 cycles and paired-read sequencing conditions. Each sample was sequenced at a depth of at least 5 × 10^7^ reads.

### Sequencing data analyses

Fastq files were mapped to the mm10 mouse genome assembly using Bowtie2 [78] with default parameters. Tag directories were made using the makeTagDirectory option in the HOMER suite [79]. For loci visualization, BedGraph files were generated for each biological replicate (using the makeUCSCfile option in the HOMER suite). For normalization, total number of reads in BedGraph files was set to 10^7^. BedGraph files were then converted to tdf files by the integrated genome browser (IGV) [80]. Then, selected gene loci were visualized using IGV.

### Differential gene expression

Differential gene expression was evaluated by DESeq2 [81] via the HOMER suite under default parameters. Genes were determined as differentially expressed between 2 conditions if they pass these cutoffs: FC ≥1.5, adjusted *p*-value ≤0.05.

### t-distributed stochastic neighbor embedding (t-SNE)

t-SNE was performed by using the Rtsne package (R version 4.2.1). The analyzed values are log2(RPKM+1). Genes with RPKM < 0.5 were excluded.

### k-means clustering

In the fasting-induced analysis, all genes induced by fasting (compared to at least one of the fed states) were included in the clustering analysis. In the fasting-repressed analysis, all genes repressed by fasting (compared to at least one of the fed states) were included in the clustering analysis. The normalized tag counts of each gene were used for the analyses. Morpheus (https://software.broadinstitute.org/morpheus) was used to cluster genes under these parameters: k = 3; metric—one minus Pearson correlation; maximum iterations– 1,000. Blue—minimum value of the gene; Red–maximum value of each gene (minimum and maximum values of each gene are set independently to other genes).

### ATAC-seq and ChIP-seq analyses

Peak-calling was performed by MACS2 (narrowPeak option) [82]. In ChIP-seq, the DKO sample was used as the control for MACS2. Differential enhancer activity was measured by DESeq2 (FC ≥1.5, adj. *p*-value ≤0.05). Genomic annotations were made by the HOMER suite (annotatePeaks option, parameter -annStats). Nearest gene analyses were performed by the HOMER suite (annotatePeaks option).

### Bivariate genomic footprinting (BaGFoot)

BaGFoot was performed as described [57]. Briefly, the 3 replicates from each condition (Adlib, Refed_24h) were merged into a single BAM file. Accessible sites were called for each BAM file using MACS2. The FPD and FA were calculated for each known motif across all accessible sites. The difference (Δ) between Adlib and Refed_24h were calculated and plotted on the bag plot.

### De novo motif enrichment analysis

To unbiasedly detect enriched motifs, we performed a de novo motif enrichment analysis using the findMotifsGenome option in HOMER (parameter -size given). The entire enhancer landscape (all ATAC accessible sites across all conditions) was used as background to account for possible sequence bias. Using the entire enhancer landscape as background ensures that prevalent motifs appearing across liver enhancers will not be falsely detected as specifically enriched in the examined subset of enhancers. In motif enrichment analyses of total ATAC accessible sites, the background was automatically selected by HOMER to account for GC bias and other sequence biases.

### Aggregate plots and box plots

Tag density of ATAC or LXRα ChIP signal around ATAC site center or transcription start site (TSS) were analyzed using the HOMER suite. In aggregate plots, the tag count (averaged across all sites) per site per bp was calculated using the HOMER suite (annotatePeaks, option -size 8,000 -hist 10). In box plots, tag count +/− 200 bp around the site center (averaged across all sites) was calculated using the HOMER suite: annotatePeaks, option -size 400 -noann. In both aggregate plots and box plots, the data is an average of all 3 replicates. In all box plots, the 10 to 90 percentiles are plotted.

### Analysis of data from the literature

GW3965-induced genes were determined by analyzing previously published data [60] as were rhythmic genes dictated by the circadian clock [46]. Differential gene expression was evaluated by DESeq2 [81] via the HOMER suite under default parameters. Genes were determined as differentially expressed between 2 conditions if they pass these cutoffs: FC ≥1.5, adjusted *p*-value ≤0.05.

### Statistical analyses

All conditions in all of the described experiments were performed in at least 3 biological replicates. Error bars represent standard deviation of biological replicates. In pairwise comparisons, statistical significance was determined by a two-tailed, unpaired *t* test. In comparisons of 3 or more groups, ordinary one-way ANOVA was performed with post hoc analyses made via Holm–Sidak or Dunnett’s tests as specified in the figure legends. In RNA-seq and ATAC-seq experiments, DESeq2 was used to determine statistical significance. *P ≤ 0.05, **P ≤ 0.01, ***P ≤ 0.001, ****P ≤ 0.0001, ^ns^ *P* > 0.05. Further details about statistical analyses are described in figure legends.

## Supporting information

S1 Fig Minimal overlap between refeeding-induced genes and clock-controlled genes. Evaluation of Refed-induced genes vs. clock-controlled genes show a distinct and nonoverlapping set of genes. The effect of zeitgeber time (ZT) on gene expression was measured in ZTs matching the ZTs at which Refed_3h and Refed_10h samples were collected (ZT4 and ZT11, respectively). The partial overlap suggests that most refeeding-induced genes are induced due to refeeding per se rather than due to circadian rhythm.
(TIF)

S2 Fig Overshoot is evident also in longer refeeding periods. **(A)** Plasma triglycerides and cholesterol were quantified in Adlib and Refed_24h mice, showing no change in both parameters following refeeding. **(B)** Refed groups underwent 24 h of fasting followed by refeeding. Livers were collected 24 h, 72 h, and 1 week after reintroduction of food. The expression of representative LIPO and AID genes was measured via quantitative PCR (qPCR), showing that for some genes, overshoot is evident 72 h following refeeding. **(C)** Liver triglycerides were measured in the livers collected in B, showing a slight (statistically insignificant) increase in hepatic triglycerides following refeeding. **(D)** Refed groups underwent 8 h of fasting followed by refeeding. Livers were collected 24 h and 72 h after reintroduction of food. The expression of representative LIPO and AID genes was measured via qPCR, showing no gene overshoot following refeeding.—All individual data points are presented ± SD; *P ≤ 0.05, **P ≤ 0.01, ***P ≤ 0.001, ****P ≤ 0.0001; by two-tailed unpaired *t* test (A) or ordinary one-way ANOVA followed by Dunnett’s post hoc analysis (B–D). Numerical values for this figure are detailed in S1 Data.
(TIF)

S3 Fig Most liver ATAC sites show enhancer characteristics. **(A)** Genomic annotations of ATAC accessible sites show that the vast majority of liver accessible sites are not promoter-proximal. Promoter-proximal regions were defined as −1 kb to +0.1 kb from the transcription start site (TSS). **(B)** Motif enrichment analysis of total ATAC accessible sites shows enrichment of liver lineage-determining factors known to bind hepatic enhancers, suggesting these sites are largely comprised of liver enhancers.
(TIF)

S4 Fig LXRα directly binds its affected enhancers. **(A)** Liver qPCR analysis of WT and double knockout (DKO) mice show that DKO mice do not express *Nr1h2* or *Nr1h3*. All individual data points are presented ± SD; *P ≤ 0.05, **P ≤ 0.01, ***P ≤ 0.001, ****P ≤ 0.0001; by two-tailed unpaired *t* test. **(B)** Analysis of ATAC-seq found that LXR-increased enhancers in the livers of refed mice are enriched for the LXR motif. **(C)** Analysis of LXRα ChIP-binding sites shows they are enriched for the LXR motif. **(D)** ChIP-PCR experiment measuring LXRα binding near LIPO genes. LXRα binding is constitutive and is evident in all conditions: Adlib, Fasted, Refed_24h. Background signal is shown in the control–LXRα ChIP in livers from DKO mice. In all samples, the ChIP signal was normalized to input.—Numerical values for this figure are detailed in S1 Data.
(TIF)

S1 Table Differentially regulated genes and refeeding-induced genes. (XLSX)

S2 Table Gene clustering and pathway enrichment analysis. (XLSX)

S3 Table LIPO-CHOL-AID genes. (XLSX)

S4 Table Enhancer BaGFoot and motif enrichment analyses. (XLSX)

S5 Table LXR-regulated genes. (XLSX)

S6 Table LXR-regulated ATAC-seq sites and LXR ChIP-seq binding sites. (XLSX)

S1 Data Numerical values of graphs. (XLSX)

## Abbreviations

ChIP: chromatin immunoprecipitation
ChREBP: carbohydrate response element-binding protein
DKO: double knockout
DSG: disuccinimidyl glutarate
FA: flanking accessibility
FC: fold change
FPD: footprint depth
LRH-1: liver receptor homolog 1
LXR: liver X receptor
PBS: phosphate-buffered saline
qPCR: quantitative PCR
SREBP: sterol regulatory element-binding protein
TF: transcription factor
ThR: thyroid hormone receptor
t-SNE: t-distributed stochastic neighbor embedding
TSS: transcription start site
USF-1: upstream stimulatory factor 1
WT: wild type
XBP-1: X-box binding protein 1
ZT: zeitgeber time
