# Supplementary figures and images for: LXR-dependent enhancer activation regulates the temporal organization of the liver’s response to refeeding leading to lipogenic gene overshoot
Source: PLoS Biol. 2024 Sep 12;22(9):e3002735. doi: 10.1371/journal.pbio.3002735 (PMC11379474; doi:10.1371/journal.pbio.3002735)

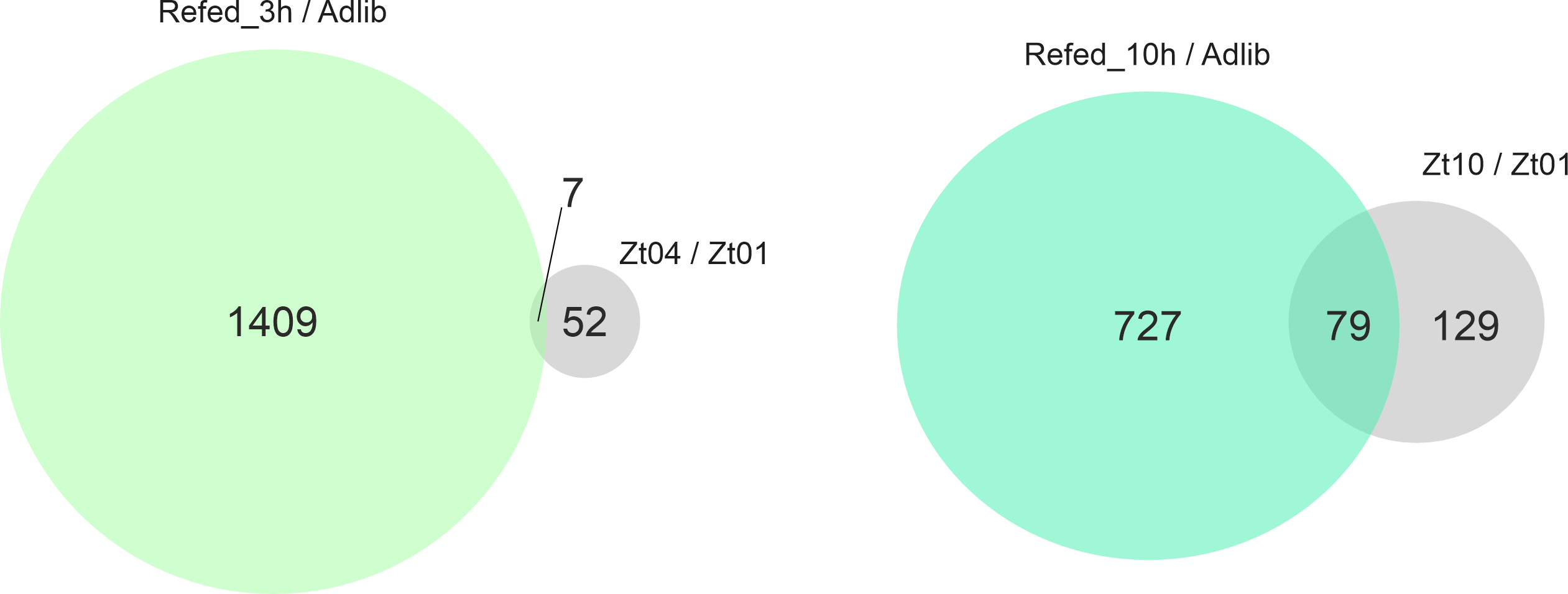

Supplement: S1 Fig — Evaluation of Refed-induced genes vs. clock-controlled genes show a distinct and nonoverlapping set of genes. The effect of zeitgeber time (ZT) on gene expression was measured in ZTs matching the ZTs at which Refed_3h and Refed_10h samples were collected (ZT4 and ZT11, respectively). The partial overlap suggests that most refeeding-induced genes are induced due to refeeding per se rather than due to circadian rhythm. (TIF) [file pbio.3002735.s001.tif]

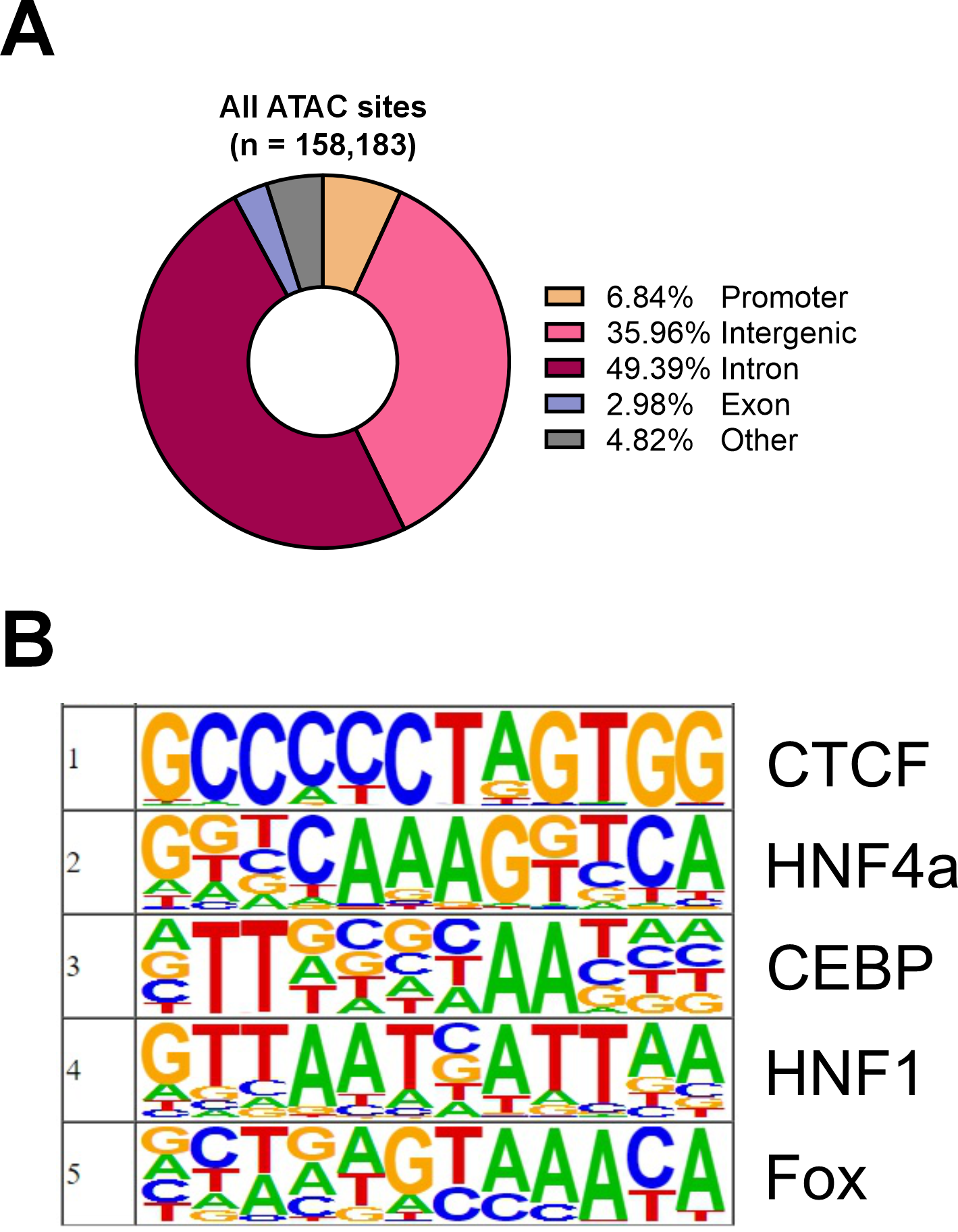

Supplement: S3 Fig — (A) Genomic annotations of ATAC accessible sites show that the vast majority of liver accessible sites are not promoter-proximal. Promoter-proximal regions were defined as −1 kb to +0.1 kb from the transcription start site (TSS). (B) Motif enrichment analysis of total ATAC accessible sites shows enrichment of liver lineage-determining factors known to bind hepatic enhancers, suggesting these sites are largely comprised of liver enhancers. (TIF) [file pbio.3002735.s003.tif]
